# A Pilot Study on the Effects of Sweet Potato Petiole and Leaf Powder on Gut Microbiota and Aging-Related Biomarkers in an Aged Microminipig Model

**DOI:** 10.3390/metabo15110713

**Published:** 2025-10-30

**Authors:** Kazuhisa Sugai, Yoshiaki Miyamoto, Toshiyuki Sato, Yoji Hakamata, Toshiyuki Murakoshi, Shou Kobayashi, Sadahiko Iwamoto, Eiji Kobayashi

**Affiliations:** 1 Institute for Integrated Sports Medicine, Keio University School of Medicine, 35 Shinano-machi, Shinjuku-ku, Tokyo 160-8582, Japan; 2 Sysmex Corporation, 4-4-4 Takatsuka-dai, Nishi-ku, Kobe-shi 651-2271, Hyogo, Japan; 3 Division of Basic Science, School of Veterinary Nursing and Technology, Nippon Veterinary and Life Science University, 1-7-1 Kyonan-cho, Musashino-shi 180-8602, Tokyo, Japan; 4 Asahi Animal Hospital, 2-36-3 Higashi-Koigakubo, Kokubunji-shi 185-0014, Tokyo, Japan; 5 Kobayashi Regenerative Research Institute, LLC, 1 Chayano-cho, Wakayama-shi 640-8263, Wakayama, Japan

**Keywords:** aged, swine, miniature, ipomoea batatas, polyphenols, microbiota, flow cytometry

## Abstract

**Background/Objectives:** Although many healthy foods are used for elderly humans, there are no suitable animal models to test them. We reared microminipigs (MMPs) for over 10 years, establishing aged MMP models. Using this aged preclinical model, we evaluated the effects of a polyphenol-rich diet without sacrificing the animals. **Methods:** A polyphenol-rich diet containing sweet potato petioles and leaves was administered to the aged MMPs daily for one month. Changes in fecal microbiota and aging-related cells in the peripheral blood before and after administration were assessed. **Results:** Administration of a diet containing sweet potato petiole and leaf resulted in increased abundance of the genera *Muribaculaceae*, *Oscillibacter*, and *Desulfovibrio* and a decreased abundance of the genus *UCG-002* within the family *Oscillospiraceae*. Prediction of metabolic enzyme activity from microbiota composition identified 77 enzymes significantly altered after administration. KEGG Mapper analysis of these enzymes revealed their involvement in 27 pathways. Flow cytometry analysis of peripheral blood revealed no significant differences in the proportion of ß-galactosidase-positive cells in either group. In contrast, a significant increase in the number of Ki-67-positive cells was observed in some individuals in the treatment group. However, no significant differences in Ki-67 expression were detected after stimulation with anti-CD3/CD28 antibodies. **Conclusions:** We established an aged MMP model to evaluate the efficacy and safety of drugs and health foods in elderly humans. Using this model, sweet potato petioles and leaves were shown to have potential as candidate materials for future health food research.

## 1. Introduction

Since the beginning of the 21st century, birth rates have declined and life expectancy has increased worldwide, and population aging has accelerated markedly [1,2]. The aging population is expected to continue to grow in the future [3,4], raising concerns about the increasing strain on medical resources owing to the decline in the working-age population [1]. Furthermore, as the population continues to age, aging itself has become a major risk factor for chronic diseases, and aging-related diseases and multimorbidity have led to rising healthcare expenditure per person, placing increasing strain on healthcare financing systems [2,5]. Therefore, interventions targeting the aging process are increasingly being recognized as crucial for preventing or inhibiting the progression of chronic diseases [6,7].

In research on health and anti-aging in the elderly, clinical trials on pharmacological interventions have been conducted in the elderly [8]. However, it is often difficult to conduct clinical trials on elderly patients because of their decline in physical function and the presence of comorbidities [9,10]. Additionally, there are difficulties in obtaining informed consent from patients with cognitive impairment, which may limit their participation in clinical trials [11].

Various animal species have been used in aging research, including rodents (mice and rats), primates (non-human primates, NHPs), carnivorans (dogs and cats) [12], birds, and fish [13]. Among them, NHPs have been widely used as animal models in biomedical research because of their genetic and physiological similarities to humans. Although NHPs have been extensively used in aging research, their use has been limited in recent years owing to their long lifespans and high associated costs. On the other hand, mice and rats are the most commonly used animals in aging research due to their well-characterized genetic backgrounds, known physiological traits, ease of handling, shorter lifespans, and lower research costs compared to NHPs. However, validating the Human Equivalent Dose in rodents is challenging [14,15].

Pigs share many anatomical and physiological similarities with humans and have attracted attention as experimental animals, and translational research using swine models has been actively developed [16,17]. Domestic pigs may be used depending on the research purpose; however, miniature pigs bred for smaller sizes and standardized strains are mainly used [18,19]. One of the authors, E.K., has been conducting research and development on the microminipig (MMP), the world’s smallest experimental minipig, and has managed a colony of MMPs continuously for over 10 years, allowing them to age naturally, in order to conduct this study. Such long-term rearing and natural aging of pigs is feasible with MMPs, due to their small body size and the cost-effectiveness of rearing these animals [20,21].

This study evaluated the effects of the long-term administration of sweet potato petioles and leaves on aged MMPs. To assess these effects in a minimally invasive manner, gut microbiota analysis and evaluation of senescence-associated cells in the peripheral blood were performed. Polyphenols found in most plants are expected to be utilized as health foods because of their antioxidant effects and potential to ameliorate arteriosclerosis, diabetes, and obesity [22,23,24,25]. Sweet potato (*Ipomoea batatas* L.), an edible crop belonging to the family *Convolvulaceae* and order *Solanales*, originated in Central and South America more than 5000 years ago and is consumed worldwide [26]. Although the tuberous roots of sweet potatoes are primarily consumed, the petioles and leaves, which are usually discarded, are also rich in dietary fiber, polyphenols, vitamins, and minerals, and have been reported to have antioxidant [27,28], anti-obesity [29,30], antidiabetic [31,32,33,34], anti-inflammatory [35,36], and anti-aging effects [37].

In this study, we first established a colony of aged experimental MMPs as a model to evaluate the efficacy and safety of drugs and health foods for the elderly. To verify the applicability of this model, we conducted a minimally invasive pilot study using a diet containing sweet potato petioles and leaves, demonstrating its potential to contribute to the development of health foods for the elderly, while also maintaining lifelong breeding of the animals.

## 2. Materials and Methods

### 2.1. Animals

The microminipigs (MMPs) used in the experiments were purchased from Fuji Micra Co., Ltd. (Shizuoka, Japan). Prior to the start of this study, the animals were housed for over 10 years in an environment maintained at 20 ± 5 °C, 55 ± 35% humidity, and a 11-h:13-h light-dark cycle, receiving 360 g of standard diet (formulated feed for medium pigs, Multirack, CHUBU SHIRYO Co., Ltd., Nagoya-shi, Aichi, Japan) once daily in the morning, with both feed and water available ad libitum, and allowed to age naturally. Two male and three female aged MMPs, aged 8–13 years, were used in this study (Table 1).

Generally, the average lifespan of pigs is approximately 20 years, and their maximum expected lifespan is 27 years [17,38]. However, domestic pigs are typically slaughtered early due to their rapid growth, resulting in a much shorter average lifespan of approximately 1.8 years [38]. In previous reports, the oldest aged pigs were around 10 years old, with most being approximately 8 years old [21,39,40,41,42,43]; therefore, the MMPs used in this study are regarded as aged.

### 2.2. Experimental Protocols

In this study, we used the aboveground stems (vines) of the Japanese sweet potato cultivar Silk Sweet cultivated in Kokubunji, Tokyo, Japan. After harvesting, the vines were sun-dried and separated into petioles and stems. The petioles and leaves were pulverized using a grinder (Ikemoto Rika Kogyo Co., Ltd., Kawasaki-shi, Kanagawa, Japan), and the resulting powder, which was sieved through a 1-mm mesh, was used in the experiment. The polyphenol content in a mixed powder of petioles and leaves of a Japanese sweet potato cultivar, Silk Sweet, was estimated using the Folin–Ciocalteu method. The polyphenol content was 3720 mg/100 g gallic acid equivalents (GAE) in Silk Sweet, indicating that Silk Sweet had a higher polyphenol content than major vegetable, fruits, and other foods [44]. The effects and toxicity of the sweet potato petiole and leaf powder used in this study were verified in a previous study using small animals (gerbils) at the Nippon Veterinary and Life Science University (Permit No. 2024 K-62).

Aged MMPs were divided into a control group (N = 2) that was fed a standard diet (360 g/day) and a potato group (N = 3) that was fed a diet containing 10% powdered sweet potato petioles and leaves (324 g/day standard diet + 36 g/day powdered sweet potato petioles and leaves; total 360 g/day). These diets were administered daily for one month. Fecal and peripheral blood samples were collected before (Pre) and one month after (Post) the initiation of sweet potato petiole and leaf administration. The health condition of the animals was monitored daily through observation of activity, appearance, fecal condition, and remaining feed. Humane endpoints were predefined; in cases of distress or moribund condition, euthanasia was performed under deep isoflurane anesthesia via exsanguination from the axillary or femoral vessels. This study was approved by the Institutional Animal Care and Use Committee of Clino Corp. (Sendai-shi, Miyagi, Japan) in accordance with the standards of the Association for Assessment and Accreditation of Laboratory Animal Care International (Permit No. Clino25005, Permit Date: 1 February 2025). All sections of the study adhered to the ARRIVE guidelines for animal research [45].

### 2.3. Gut Microbiota Analysis from Fecal Samples

Fecal samples collected before (Pre) and one month after (Post) the sweet potato petiole and leaf administration were homogenized in a 4 M guanidinium thiocyanate buffer, and DNA was extracted. The 16S rDNA region from each fecal DNA sample was amplified using dual-index primers, and sequencing was performed using the MiSeq platform (Illumina, Inc., San Diego, CA, USA) to generate FASTQ files. The FASTQ files were processed using QIIME2 version 2023.5 [46], and taxonomic classification was performed with reference to the SILVA database version 138. From the resulting data, α-diversity (Chao1 index) and ß-diversity (principal coordinate analysis using both weighted and unweighted UniFrac distances) were calculated. Taxonomic composition at the genus level (level 7) was analyzed, and LEfSe (Linear Discriminant Analysis Effect Size) analysis [47] was performed to identify bacterial taxa that were significantly altered following sweet potato petiole and leaf administration. In addition, metabolic pathway prediction was conducted using PICRUSt2 version 2021.2 [48] and the ggpicrust2 package in R [49], with functional annotation based on the Kyoto Encyclopedia of Genes and Genomes (KEGG) database. Statistical analyses were conducted using R version 4.3.2, and differences before and after treatment were assessed using paired *t*-tests or Wilcoxon signed-rank tests with a significance threshold of *p* < 0.05. For multiple comparisons, a false discovery rate (FDR) < 0.2 was considered statistically significant. To minimize batch effects, all sample collection, DNA extraction, library preparation, and sequencing were performed on the same day by the same operator. Quality control of sequencing data was also conducted to ensure data reliability.

### 2.4. Analysis of Senescence-Associated Cells in Peripheral Blood

For analysis of senescence-associated cells in peripheral blood, 5 mL of peripheral blood was collected from the subclavian veins of the animals before (Pre) and one month after (Post) the sweet potato petiole and leaf administration. The collected blood was diluted two-fold with PBS (FUJIFILM Wako Pure Chemical Corp., Osaka, Japan), and peripheral blood mononuclear cells were isolated by density gradient centrifugation using Ficoll-Paque™ PLUS (Cyteva, Marlborough, MA, USA) at 400× *g*, 24 °C for 30 min. The collected mononuclear cell layer was washed with 10 volumes of PBS and centrifuged again at 400× *g*, 24 °C for 5 min. After removing the supernatant, the cells were resuspended in RPMI 1640 medium (FUJIFILM Wako Pure Chemical Corp., Osaka, Japan) containing 10% FBS (Thermo Fisher Scientific, Waltham, MA, USA).

To identify senescent cells, ß-galactosidase (ß-gal) staining was performed using the Cellular Senescence Detection Kit—SPiDER-ßGal (Dojindo Molecular Technologies, Inc., Kumamoto, Japan). Specifically, the cell suspension was centrifuged at 400× *g*, 24 °C for 5 min, and the supernatant was removed. The cells were then resuspended in 1 mL of HBSS (FUJIFILM Wako Pure Chemical Corp., Osaka, Japan), centrifuged again under the same conditions, and the supernatant was discarded. Subsequently, the cells were resuspended in 1 mL of Bafilomycin A1 working solution and incubated at 37 °C for 1 h. Next, 1 mL of SPiDER-ßGal working solution was added, and the mixture was incubated at 37 °C for 30 min. After staining, the cells were centrifuged (400× *g*, 24 °C, 5 min), the supernatant was removed, and the cells were resuspended in 10% FBS/RPMI 1640 medium, followed by another centrifugation. Finally, the cells were resuspended in 1% BSA/PBS. APC-conjugated anti-CD3 antibody (BD Biosciences, Franklin Lakes, NJ, USA) was added to the stained cells and incubated on ice for 15 min. After two washes with 1% BSA/PBS (400× *g*, 24 °C, 5 min), the cells were resuspended again in 1% BSA/PBS. Flow cytometric analysis was performed using a BD FACSLyric flow cytometer (BD, Franklin Lakes, NJ, USA), and data were analyzed using the FlowJo v10 software platform (FlowJo LLC., Ashland, OR, USA).

Lymphocyte proliferative capacity was assessed by Ki-67 staining using the BD Pharmingen Transcription Factor Buffer Set (BD, Franklin Lakes, NJ, USA). Specifically, the cell suspension was centrifuged at 400× *g*, 24 °C for 5 min, then resuspended in 1× Fix/Purm solution and incubated at 4 °C for 40 min. After two washes with 1× Perm/Wash buffer (400× *g*, 24 °C, 5 min), the supernatant was removed, and cells were incubated with APC-conjugated anti-Ki-67 antibody (BD, Franklin Lakes, NJ, USA) diluted in 1× Perm/Wash buffer at 4 °C for 40 min. After two additional washes, cells were resuspended in 1% BSA/PBS medium. Flow cytometric analysis was performed using BD FACSLyric, and the data were analyzed using FlowJo v10.

A stimulation assay with anti-CD3/CD28 antibodies was performed on cryopreserved lymphocytes. Specifically, 96-well flat-bottom plates were coated with anti-CD3 antibody (Novus Biologicals, Centennial, CO, USA) diluted in PBS (final concentration: 1 μg/mL) and incubated for 2 h at 37 °C in a 5% CO_2_ incubator. After two washes with PBS, the cell suspension was added to each well. Anti-CD28 antibody (MyBioSource, San Diego, CA, USA) diluted in 10% FBS/RPMI 1640 medium (final concentration: 1 μg/mL) was then added, and the plate was centrifuged at 200× *g* for 2 min at 24 °C to facilitate cell contact with the well bottom. Cells were cultured for 6 d at 37 °C in a 5% CO_2_ incubator. After incubation, the cells were harvested, and Ki-67–positive cells were detected.

Statistical analyses of these experimental results were performed using paired *t*-tests, with differences considered significant at *p* < 0.05.

## 3. Results

There were no abnormalities in the animals’ health conditions, including activity, appearance, and fecal conditions, during the experimental period, and no deaths occurred.

### 3.1. Gut Microbiota Changes Induced by Sweet Potato Petiole and Leaf

16S rDNA V3-V4 region sequencing using fecal DNA collected before and after the administration of sweet potato petioles and leaves yielded 102,702 ± 11,321 non-chimeric reads per sample. Based on these results, statistical analyses were conducted to evaluate the changes in the gut microbiota before and after the administration of sweet potato petioles and leaves.

**Figure 1:**
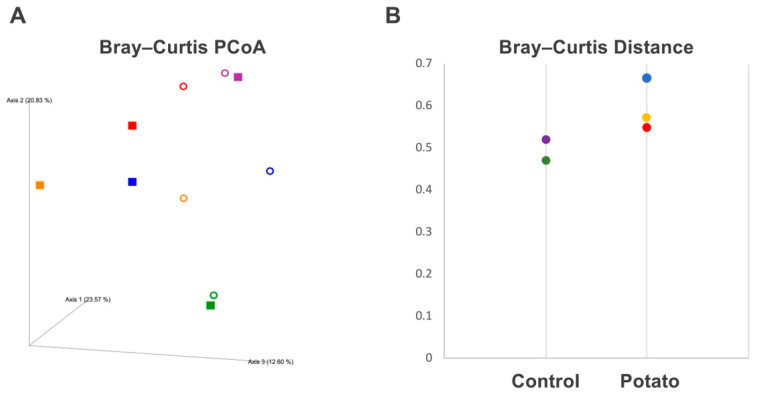
ß-diversity Analysis. (**A**) Bray–Curtis PCoA plot. The open circles (○) indicate values before the administration of sweet potato petiole and leaf or the control diet, while the squares (■) indicate values after administration. (**B**) Bray–Curtis Distance. Bray–Curtis distances before (Pre) and one month after (Post) the administration of sweet potato petiole and leaf or the control diet were plotted for each individual within each group. Red, yellow, and blue indicate individual data points in the potato group, whereas purple and green represent those in the control group.

No significant changes were observed in the α-diversity analysis, including observed OTUs, Chao1, Shannon index, and Faith’s phylogenetic diversity, either between the control and potato groups or between pre- and post-administration. In ß-diversity analysis, principal coordinate analysis (PCoA) based on the Bray–Curtis distance revealed that in the potato group, the distances between pre- and post-administration samples from the same individual were greater than those in the control group (Figure 1A). Although the Bray–Curtis distances tended to be greater in the potato group than in the control group, this difference was not statistically significant (Figure 1B).

**Figure 2:**
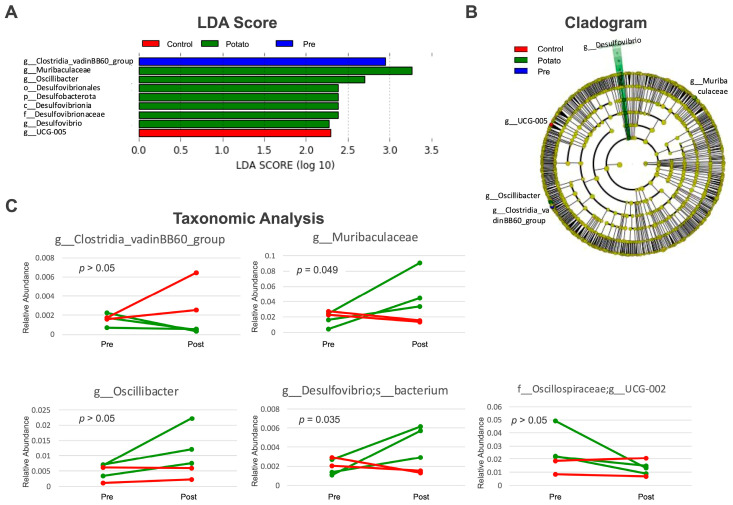
LEfSe Analysis. (**A**) LDA score (log10) bar plot. (**B**) Cladogram. Bacterial species enriched after control diet administration (red), pre-administration of sweet potato petiole and leaf (blue), and post-administration of sweet potato petiole and leaf (blue) are shown at the identified taxonomic level. g; genus, f; family, o; order, c; class, p; phylum. (**C**) Taxonomic analysis. Pairwise plots show the relative abundance of bacterial species that exhibited significant changes as suggested by LEfSe analysis, before (Pre) and after (Post) the administration of the control diet (red) or sweet potato petiole and leaf (green). *p*-values indicate the results of paired *t*-tests comparing values pre- and post-administration of sweet potato petiole and leaf.

In the LEfSe analysis using the individual as a subclass variable, the administration of sweet potato petioles and leaves resulted in an increase in *Muribaculaceae* (log LDA = 3.262. *p* = 0.040), *Oscillibacter* (log LDA = 2.699, *p* = 0.048), and *Desulfovibrio* (log LDA = 2.385, *p* = 0.048), as well as a decrease in the genus *UCG-002* within the family *Oscillospiraceae* (log LDA = 2.949, *p* = 0.022), whereas the control group showed an increase in the genus *Clostridia_vadinBB60_group* (log LDA = 2.293, *p* = 0.022) (Figure 2A,B). Among these, the genera *Muribaculaceae* and *Desulfovibrio* showed a significant increase in pre- and post-administration of sweet potato petioles and leaves, as confirmed by the paired *t*-test (Figure 2C).

By predicting the activated or suppressed metabolic enzymes from the percentage of microbiota using PICRUSt, 431 enzymes were found to differ significantly (*p* < 0.05) before and after sweet potato petiole and leaf administration based on a paired *t*-test, and 77 enzymes remained significant after multiple testing corrections. Although no significantly enriched KEGG pathways were identified in the ggpicrust2 analysis, the KEGG Mapper analysis of the 77 enzymes that remained significant after multiple testing corrections revealed their involvement in 27 pathways (Table 2).

### 3.2. Senescence-Associated Cellular Changes in Peripheral Blood Induced by Sweet Potato Petiole and Leaf

**Figure 3:**
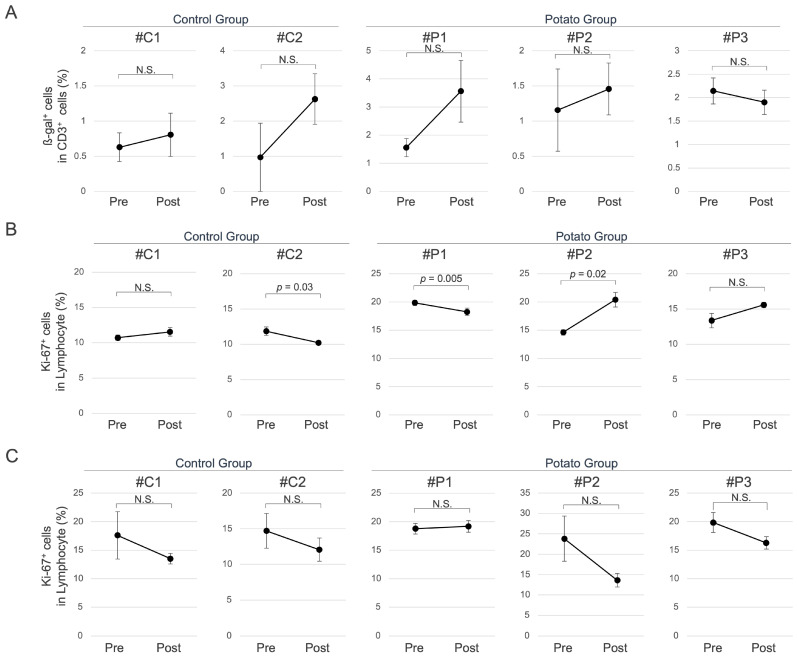
Senescence-Associated Cellular Changes in Peripheral Blood. (**A**) Proportion of ß-gal-positive cells among CD3-positive cells. (**B**) Proportion of Ki-67-positive cells among lymphocytes. (**C**) Proportion of Ki-67-positive cells among lymphocytes after stimulation with anti-CD3/CD28 antibodies. *p*-values indicate the results of paired *t*-tests comparing values before (Pre) and one month after (Post) administration of sweet potato petiole and leaf. N.S., not significant.

Given the use of aged MMPs in this study, senescent cells in peripheral blood lymphocytes were evaluated by flow cytometry in both potato and control groups. No significant differences were observed in the proportion of ß-galactosidase (ß-gal)-positive cells, a widely recognized marker of cellular senescence (Figure 3A). In contrast, a significant increase in the proportion of cells expressing Ki-67, a marker of cellular proliferation, was observed in some individuals of the potato group (Figure 3B). Furthermore, to assess lymphocyte responsiveness, the proportion of Ki-67-positive cells was measured following stimulation with anti-CD3 and anti-CD28 antibodies; however, no significant differences were observed between the groups (Figure 3C).

## 4. Discussion

As global population aging has become an increasingly serious social issue, it is becoming increasingly important to prevent or inhibit aging because it is a major risk factor for various chronic diseases [6,7,50,51]. However, clinical trials involving elderly subjects are limited, making the use of experimental animals in aging research more common. In previous reports, the oldest aged pigs were around 10 years old, with most being approximately 8 years old [21,39,40,41,42,43]. Although the oldest individual used in this study was 13-year-old, most were 8–9 years old; therefore, we defined aged MMPs as those between 8 and 13 years old.

Sweet potatoes are divided into four parts: tuberous roots, stems, petioles, and leaves, with the tuberous roots being primarily consumed. Although parts other than the tuberous root are usually discarded, they are rich in dietary fiber, polyphenols, vitamins, and minerals and have gained attention because of their antioxidant [27,28], anti-obesity [29,30], antidiabetic [31,32,33,34], anti-inflammatory [35,36], and anti-aging effects [37]. Therefore, using the discarded petioles and leaves of sweet potatoes as a healthy food is considered valuable from the perspective of Sustainable Development Goals.

Analysis of the gut microbiota from the feces of Aged MMPs administered to sweet potato petioles and leaves showed a tendency toward greater divergence in Bray–Curtis distance in ß-diversity analysis compared to those in the control group. The Bray–Curtis distance is an index of ecological similarity based on the abundance of each microbial species, and the results suggest that the administration of sweet potato petioles and leaves altered the relative abundance of certain species. Consistently, LEfSe analysis revealed an increase in the genera *Muribaculaceae*, *Oscillibacter*, and *Desulfovibrio*, and a decrease in the genus *UCG-002* (family *Oscillospiraceae*) following the administration of sweet potato petioles and leaves. Among these, increases in the genera *Muribaculaceae* and *Desulfovibrio* were validated using a paired *t*-test. Bacteria of the genus *Muribaculaceae* cooperate with *Bifidobacterium* and *Lactobacillus* to produce short-chain fatty acids from plant-derived fibers, contributing to beneficial effects in inflammatory bowel disease, obesity, and type 2 diabetes [52]. Therefore, prebiotics that promote an increase in *Muribaculaceae* bacteria have been studied, and in addition to plant-derived fibers, polyphenols have also been reported to contribute to their proliferation [52]. The petioles and leaves of sweet potatoes, which are rich in both dietary fibers and polyphenols, may serve as ideal prebiotic materials. *Desulfovibrio* species are also involved in short-chain fatty acid synthesis; however, they are known to influence immune signaling regulation by catalyzing hydrogen sulfide (H_2_S) production [53]. H_2_S can exert immunosuppressive effects by negatively regulating immune signaling pathways such as NLRP3; however, it can also activate immune responses by positively modulating MAPK and ERK-NF-κB signaling pathways. Therefore, there are conflicting reports regarding its involvement in the aforementioned diseases and carcinogenesis [53]. PICRUSt analysis did not reveal any changes in the pathways related to sulfate metabolism; however, enzymes associated with butanoate metabolism showed significant alterations. These results suggest that sweet potato petiole and leaf administration may promote short-chain fatty acid synthesis, potentially exerting anti-inflammatory effects and improving glucose and lipid metabolism.

The measurement of senescence markers in the peripheral blood lymphocytes of aged MMPs administered to sweet potato petioles and leaves revealed no significant changes in senescent cells due to the administration. However, increased lymphocyte proliferation has also been observed in some individuals. In these individuals, neither a decrease in ß-gal-positive cells nor an improvement in stimulation responsiveness was observed. Therefore, although this does not directly indicate a reduction in senescent cells, the administration of sweet potato petioles and leaves may induce an increase in highly proliferative lymphocytes in peripheral blood.

Short-chain fatty acids have been reported to modulate MAPK pathways and regulate inflammation [54]. The MAPK and NF-κB pathways play central roles in inflammation, and therapeutic strategies targeting these pathways have been studied [55]. Moreover, aging is associated with dysregulation of MAPK/ERK–NF-κB signaling and delayed immune responses [56,57]. In this study, increases in gut bacteria associated with short-chain fatty acid production may have influenced peripheral aging-related biomarkers via these signaling pathways. However, given that these pathways were not directly assessed, we acknowledge the limitation and have added this discussion.

This study has several limitations. Although we have compared the number of senescent cells in the peripheral blood of young and aged MMPs in a pilot study, the sample size was limited because aged MMPs require long-term care and involve substantial management costs, making them extremely valuable resources, and we have not yet obtained sufficient data to determine the age at which senescent cells significantly increase. Therefore, in the present study, we focused on the changes in the number of senescent cells in the peripheral blood of aged MMPs before and after the administration of sweet potato petiole and leaf powder; however, the sample size of aged MMPs was also limited in this study. Consequently, the generalizability of the findings is also limited. In future studies, it is necessary to increase the sample size and perform more detailed analyses. Although high intake of sweet potato petioles has been reported to be non-toxic [58], and our previous study on gerbils using a diet containing 10% sweet potato petioles and leaves (the same composition as the diet in this study) also found no toxicity, these findings were observed only in rodents. Therefore, appropriate dosages for pigs need to be investigated. We observed the animals’ condition–including activity levels, appearance, fecal conditions, and feed residue–at least once daily. However, blood biochemical parameters were not examined in this study, and future research should include analyses of metabolic indicators. Finally, in this study, the effects of sweet potato petioles and leaves were evaluated only one month after administration. Future studies should evaluate the effects during the acute phase immediately after administration and during the chronic phase after longer-term administration.

In aging research, the use of experimental animals with rapid growth and short lifespans allows efficient study progression. However, similarities to humans differ depending on the animal species. Moreover, many major age-related diseases in humans rarely occur spontaneously in experimental animals, and animal models that develop aging-related diseases at a young age often differ substantially in background and disease progression from those observed in elderly humans [59,60]. Therefore, caution should be exercised when extrapolating results from experimental animals to humans [12], and this study is no exception. A comprehensive understanding of human aging and age-related diseases can be achieved by adopting multifaceted approaches in various organisms.

## 5. Conclusions

This is the pilot study to establish a colony of aged MMPs, including 13-year-old individuals, which may serve as a useful model for aging research. We demonstrated that an aged MMP model can potentially contribute to the development of healthy foods for the elderly while maintaining lifelong breeding of the animals. Furthermore, sweet potato petioles and leaves administered to aged MMPs were shown to possess anti-inflammatory effects and improve glucose and lipid metabolism. Although sweet potato petioles and leaves are generally discarded, the results of this study suggest that they could serve as candidate materials for future health food research.

## Figures and Tables

**Table 1:** Age, Sex, and Allocation of Animals.

ID	Age	Sex	Group
#C1	8 years old	Female	Control
#C2	9 years old	Male	Control
#P1	8 years old	Female	Potato
#P2	13 years old	Female	Potato
#P3	9 years old	Male	Potato

**Table 2:** KEGG mapper analysis result of enzymes showing significant changes identified by PICRUSt analysis.

KEGG ID	Pathway Name	Mapped Number
ko01100	Metabolic pathways	52
ko00195	Photosynthesis	27
ko01110	Biosynthesis of secondary metabolites	14
ko00190	Oxidative phosphorylation	11
ko00860	Porphyrin metabolism	7
ko00910	Nitrogen metabolism	3
ko02010	ABC transporters	3
ko01240	Biosynthesis of cofactors	3
ko00130	Ubiquinone and other terpenoid-quinone biosynthesis	2
ko00906	Carotenoid biosynthesis	2
ko00900	Terpenoid backbone biosynthesis	2
ko00770	Pantothenate and CoA biosynthesis	2
ko01232	Nucleotide metabolism	2
ko01054	Nonribosomal peptide structures	1
ko02020	Two-component system	1
ko00650	Butanoate metabolism	1
ko01230	Biosynthesis of amino acids	1
ko00230	Purine metabolism	1
ko01250	Biosynthesis of nucleotide sugars	1
ko00240	Pyrimidine metabolism	1
ko00660	C5-Branched dibasic acid metabolism	1
ko00290	Valine, leucine and isoleucine biosynthesis	1
ko00410	beta-Alanine metabolism	1
ko03060	Protein export	1
ko01210	2-Oxocarboxylic acid metabolism	1
ko00541	Biosynthesis of various nucleotide sugars	1
ko00970	Aminoacyl-tRNA biosynthesis	1

## Data Availability

The data supporting the findings of this study are available from the author upon request.
